# The associations between neighborhood walkability attributes and objectively measured physical activity in older adults

**DOI:** 10.1371/journal.pone.0222268

**Published:** 2019-09-06

**Authors:** Bo-I Chen, Ming-Chun Hsueh, Ru Rutherford, Jong-Hwan Park, Yung Liao

**Affiliations:** 1 Graduate Institute of Sports Pedagogy, University of Taipei, Taipei City, Taiwan; 2 Department of Health Promotion and Health Education, National Taiwan Normal University, Taipei City, Taiwan; 3 Health Convergence Medicine Research Group, Biomedical Research Institute, Pusan National University Hospital, Busan, Korea; University of Maiduguri College of Medical Sciences, NIGERIA

## Abstract

A limited number of studies have used objective measures to examine the associations between the built environment and physical activity (PA) among older adults. This study aimed to examine geographic information systems-derived neighborhood walkability attributes and accelerometer measured PA in older adults. Data were collected from 124 older Taiwanese adults aged over 60 years (mean age: 69.9). Adjusted multiple linear regression was performed to explore the associations between five neighborhood walkability factors (population density, street connectivity, sidewalk availability, access to destinations, and public transportation) and five metrics of accelerometer-measured physical activity (total PA, moderate-to-vigorous PA, light PA, long moderate-to-vigorous PA bouts, and daily step counts). After adjusting for potential confounders, we found that greater sidewalk availability was positively associated with daily step counts in older adults (β = 0.165; 95% confidence interval: 0.006, 0.412; P = 0.043). No associations between other neighborhood environment attributes and PA metrics were observed. In conclusion, high sidewalk availability in the neighborhood may be supportive for older adults’ daily step counts. Further longitudinal research is needed to establish the causality between the built environment and objectively measured PA in older adults.

## Introduction

Physical inactivity has led to colossal costs to global healthcare systems (approximately $53.8 billion) [1]. There has been strong evidence supporting the many health benefits of physical activity in older adults, such as decreased rates of all-cause mortality, non-communicable diseases, and functional limitations, as well as improved bone fitness, better cognitive function, and a lower risk of falling [2]. Despite this, about 31.1% of the population in the world engages in sufficient physical activity [3], and physical activity levels have increased very little since 2012 [4]. In Taiwan, nearly 40% of Taiwanese older adults are physically inactive [5]. Strategies that encourage physical activity in older adults need to be developed to prevent non-communicable diseases and to minimize the burden on the healthcare system.

According to the ecological model of health behavior [6], physical activity in older adults is influenced by their surroundings, which are determined by personal, interpersonal, and physical environmental factors. Compared with individual (psychosocial)-level intervention, making the built environment more walkable can affect a larger range of habitual physical activity in older adults in a long-term effect [7]. The features of the neighborhood built environment strongly affect independence and daily activity in older adults because older adults tend to spend most of their time in the neighborhood [8]. Neighborhoods with well-developed infrastructure and easy approach to destinations and low-cost facilities may promote the accumulation of physical activity in the everyday lives of older population [8]. However, most previous studies on the associations between the neighborhood built environment and physical activity in older adults have been conducted in Western countries [9–13], examined subjects who were in an older adult unit [10,14] or focused on a specific population [12,13]. To develop an effective community-based health promotion strategy for the general population of older Asian adults, it is critical to further focus on community-dwelling older adults in an Asian context. Another major limitation is that they rely on self-report environmental and physical activity measures [15–19]. For example, two systematic reviews have indicated that a number of previous studies have focused on the associations between built environment and self-reported context-specific physical activity (i.e., leisure-time or transport context) in older adults [10,13]. Another review also reported that few studies have examined this issue using both objectively-assessed environmental and physical activity measures [9].

To inform urban design practice, further research that examines this association using objective measurement of both is needed. A geographic information system (GIS) is a method for integrating spatial information from different sources into a single scheme and then deriving precise measures of the built environment [20]. This technology allows us to better understand the walkability attributes of the neighborhoods of older adults. Moreover, accelerometers offer the opportunity to objectively measure physical activity intensity (light, moderate, and vigorous), patterns (long bouts), and daily step counts [21], which can address the limitation of recall bias in older adults [22]. Among limited studies, a meta-analysis study indicated that only two built environmental factors, walk friendly infrastructure and destination diversity (land use mix), were positively associated with objectively assessed total physical activity [9]. A better understanding of the associations between built environmental factors and objectively measured physical activity patterns is still needed for designing effective physical activity intervention for older adults. In recent years, in order to expand the range of examined geographical settings and cultures, numerous studies have been conducted in Asian countries [14,15]. However, no study has explored the associations between objectively measured environmental attributes and physical activity in Taiwan. Taipei City is the one of the fastest aging cities in Taiwan. The city’s percentage of older adults was estimated to be 16.64% in 2018. To address the rapidly aging population of Taiwan and to strengthen the evidence base and fill the research gap, this study aimed to examine the associations between GIS-derived neighborhood walkability attributes and accelerometer-determined physical activity in older adults.

## Materials and methods

### Participants and procedures

Data on healthy older adults from 28 different neighborhoods with the ability to walk unaided living in Taipei City (an urban area) were collected during April and September in 2018. Taipei City (total population estimated to be 2,678,695), the capital of Taiwan, is the one of the fastest aging cities in Asia, and its estimated percentage of older adults was 16.64% (estimated to be 445,629) at the end of April 2018. The participants who lived in Taipei City were recruited through local advertisements and broadcasts in the neighborhood. Individuals contacted the study recruiters or neighborhood representative if they were interested in participating in the study. The inclusion criteria were as follows: (1) older adults aged 60 or above, (2) able to walk independently (individuals with assistive walking devices were excluded from the study), and (3) those who were community-dwelling, healthy older adults (such as older adults were not living in institutions).

Those eligible were invited to participate and asked to respond to a structured questionnaire that consisted of items on personal attributes, health behaviors, and health status. Next, respondents participated in an on-site examination of physical performance. Finally, at the end of the on-site examination, each participant was required to wear the accelerometer for 7 consecutive days. An incentive of a convenience store voucher (worth 7 United States dollars) was provided to the participant who completed the questionnaire, on-site examination, as well as accelerometer portion of the study.

**Fig 1:**
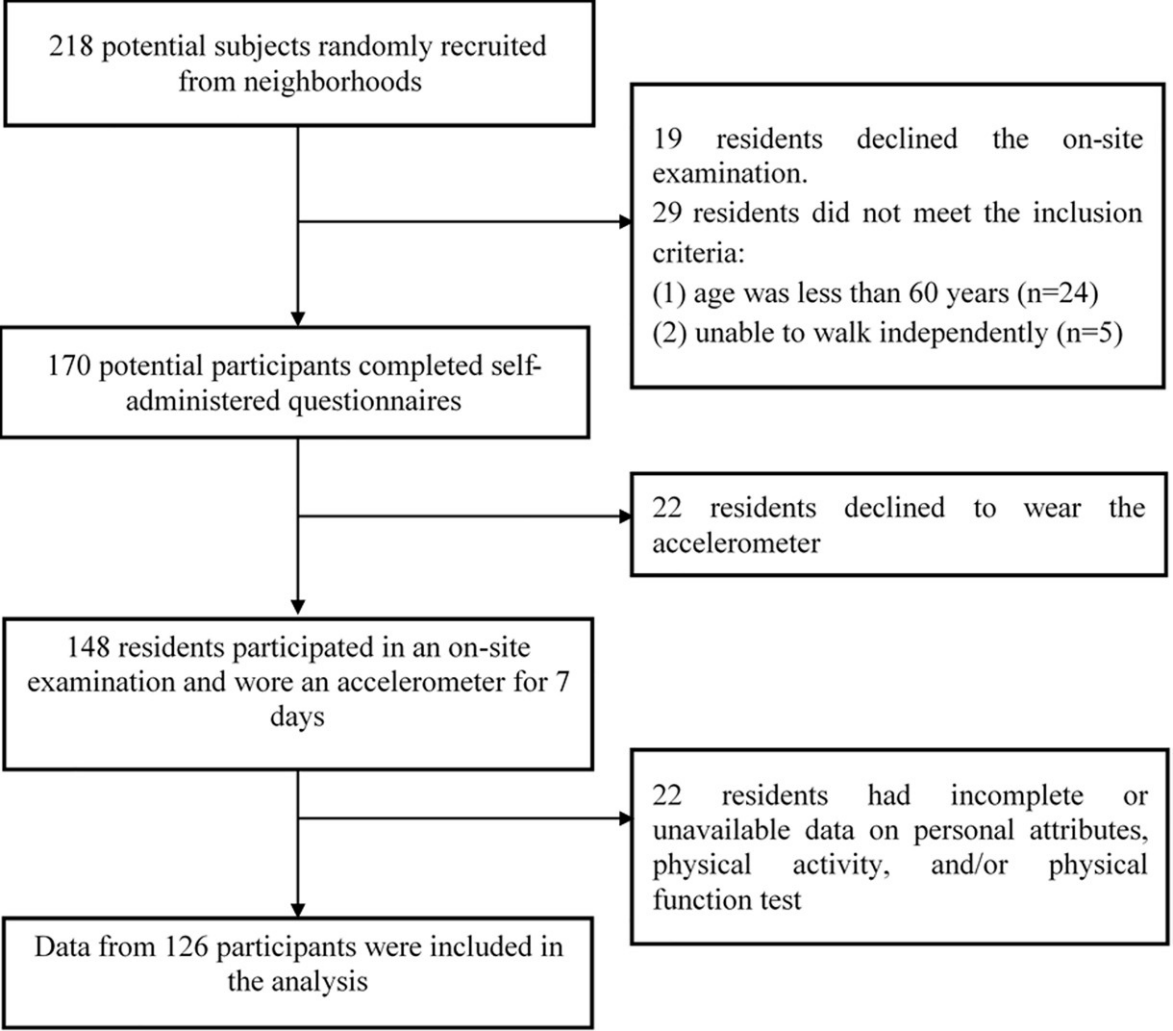
Flow of participants in this study.

A total of 218 potential participants were randomly recruited from neighborhoods in Taipei. After providing information about the study to the potential participants, 48 were excluded because they were not interested in undergoing the on-site examination (n = 19) or did not meet the inclusion criteria (n = 29). Thus, a total of 170 residents finished the questionnaire. From these, 22 declined to wear the accelerometer. A total of 148 remaining residents participated in the on-site examination and wore the accelerometer for 7 days. Twenty-two residents were excluded owing to incomplete or unavailable data on personal attributes or physical activity, or for the physical function test. Data from 126 healthy participants were included in the study’s analysis (Fig 1). Our study was conducted in accordance the 1975 Declaration of Helsinki and its subsequent revisions. Before participating in the study, each participant provided written informed consent. We obtained ethical approval from the Research Ethics Committee of the National Taiwan Normal University (REC number: 201711HM003).

### Objectively measured physical activity

Activity monitors (model wGT3X-BT; ActiGraph, Pensacola, FL, USA) were utilized to measure step counts and time spent engaged in light, moderate and vigorous-intensity physical activity. The validity and reliability of this triaxial accelerometer models have been confirmed [23]. The accelerometer records movement on 3 axes for 7 consecutive days. Data collected were processed using standard methods [24]; all analyses were conducted with data using 60-second epochs [25]. Participants were instructed to wear the monitors on their hip at all times except when engaged in water activities such as showering and swimming. For sleep time, participants reported their daily bedtime and wake time in a sleep diary. Further, we coded the sleep time by each participant's sleep onset and offset times, and used algorithms built into the ActiLife software [26, 27]. Finally, we categorized sleeping time as non-wear time, and, therefore, sleeping time was not counted as time spent sedentary. Periods of not less than 60 consecutive minutes of zero activity with allowance of up to 2 minutes of observations of limited movement were also categorized as non-wear time [23,28]. A valid day was described as having at least 10 hours (600 minutes) of monitor worn time. The results from participants with at least 3 valid days, with at least 1 weekend day was included in the present study.

Accelerometer counts that were greater than or equal to 100 counts/minute were defined as PA. Physical activity levels were classified as follows: (1) light-intensity PA (LPA): counts that were between 100 to 2,019 counts/minute; (2) moderate-to-vigorous intensity PA (MVPA): counts that were greater than or equal to 2,020 counts/minute [29]. We also calculated long MVPA bouts as periods of MVPA lasting at least 10 consecutive minutes with a 1-minute allowance below the MVPA threshold [23]. In this study, LPA, MVPA, long MVPA bouts, total PA (LPA + MVPA) and daily step counts were included as exposure variables. ActiLife software (version 6.0, Pensacola, FL, USA) was utilized to analyze accelerometer data.

### Neighborhood walkability attributes

Five neighborhood walkability attributes, which had previously been found to be associated with physical activity and walking [30], were included in this study, based on neighborhood walkability elements reported from previous studies [31,32]. These environmental walkability attributes were measured using GIS software (ArcGIS; ESRI, Redlands, CA, USA). Five environmental factors were assessed using the participants’ geocoded residential neighborhoods: population density, street connectivity, sidewalk availability, access to public transportation, and access to destinations.

Population density was calculated by the number of people per square kilometer in each participant’s geocoded residential neighborhood. The data on population density were retrieved from the National Land Surveying and Mapping Center, Ministry of the Interior, Taiwan and from the open data of the National Development Council of Taiwan [33,34]. Street connectivity was calculated by the total number of road nodes (two road links) per square kilometer; The data on street connectivity were retrieved from the National Land Surveying and Mapping Center, Ministry of the Interior, Taiwan [33]. Sidewalk availability was calculated by the sum of the areas (square meter) of the sidewalks in the participants’ residential neighborhoods. The data on the areas of the sidewalks were retrieved from the open data of the National Development Council of Taiwan [34]. Access to public transportation was calculated by the total number of mass rapid transit exits, train stations, high speed rail stations, and bus stops in a participant’s residential neighborhood. The data on public transportation were retrieved from the National Land Surveying and Mapping Center, Ministry of the Interior, Taiwan [33]. Access to destinations was calculated by the total number of 30 different destination types in each participant’s residential neighborhood, on the basis of previous studies [35,36]. The data on destinations were retrieved from the National Land Surveying and Mapping Center, Ministry of the Interior, Taiwan and from the open data of the National Development Council of Taiwan [33,34].

### Covariates

The potential confounding variables included age, gender, marital status (married or unmarried); employment (full-time job or non-full time job); education level (no tertiary education or tertiary education; tertiary education = a university or college degree or higher); living status (living alone or living with others); self-rated health (poor or good); and body mass index (BMI). Self-rated overall health was assessed with a single question on a 5-point scale (ranging from excellent to poor) from the SF-36-Item Short Form [37]. Body mass index was calculated using self-reported weight and height, and was dichotomized into non-overweight (<24 kg/m^2^) and overweight (≥24 kg/m^2^), according to Taiwanese cut-off points [38,39].

### Statistical analyses

A total of 126 older adults provided complete information for the study variables were analyzed. Independent sample t-tests were performed to compare the differences between covariates and objectively measured PA. Since a preview review indicated that not categorizing continuous environmental measures would contribute to improving the quality of future research designs [9] and the outcome variables are normally distributed, we used forced-entry multiple linear regression models for our analyses. Forced-entry multiple linear regression models adjusted for potential covariates (gender, age, marital status, educational level, employment status, living status, self-rated health, BMI, and accelerometer wear time) were conducted to examine the associations of neighborhood walkability attributes (population density, street connectivity, sidewalk availability, access to destinations, access to public transportation) with total amounts and patterns of objectively measured PA (total PA time, daily LPA time, daily MVPA time, and daily step counts). IBM SPSS 23.0 software (SPSS Inc., IBM, Chicago, IL, USA) was used for all statistical analyses in this study. The level of significance was set at p < .05.

## Results

### Participant characteristics

One hundred twenty-six older adults (men: 36, women: 90) were included in this study. The mean age of the participants was 69.9 years (standard deviation [SD] = 5.0) (Table 1). Most of the study population was aged 60 to 69 years old (59.1%), were married (65.9%), lived with others (88.9%), had no tertiary education (78.6%), was not employed (96.8%), had poor self-rated health (69.4%) and were overweight (48.4%). The mean BMI was 24.2 (SD = 3.4).

**Table 1:** Characteristics of participants.

Variables	Category	Total sample (n = 126)	
		n	(%)
Age (years)	Mean (SD)	69.9	(5.0)
	60–69	74	(59.1)
	≥70	52	(40.9)
Gender	Men	36	(28.6)
	Women	90	(71.4)
Marital status	Married	83	(65.9%)
	Unmarried	43	(34.1%)
Living status	Living with others	112	(88.9%)
	Living alone	14	(11.1%)
Educational level	Tertiary education^a^	27	(21.4%)
	No tertiary education	99	(78.6%)
Employment	Full time job	4	(3.2%)
	Non-full time job	122	(96.8%)
Self-rated health	Good	38	(30.6%)
	Poor	86	(69.4%)
BMI (kg/m^2^)	Mean (SD)	24.2	(3.4)
	Non-overweight (<24 kg/m^2^)	65	(51.6%)
	Overweight (≥24 kg/m^2^)	61	(48.4%)

SD, standard deviation; BMI, body mass index.

^a^ Tertiary education refers to a university or college degree or higher.

### Patterns of PA, step counts, and neighborhood walkability attributes

Table 2 presents the total amounts and patterns of objectively measured PA and neighborhood walkability attributes. In brief, participants accumulated a total of 316.7 (SD = 84.2) minutes per day of total PA, 292.3 (SD = 80.4) minutes per day of LPA, 24.4 (SD = 23.2) minutes per day of cumulative MVPA, and 25 (SD = 26.9) minutes per day of long-bout MVPA. They also achieved an average of 7454.6 (SD = 3404.4) steps per day. Approximately half were women (53.6%), and the majority had partners (60.1%).

**Table 2:** Total amounts and patterns of objectively measured PA, step counts, and neighborhood walkability attributes of study participants.

Accelerometer variables	Total (n = 126)	
	Mean	(SD)
Wear time (min/day)	920.5	(84.9)
Total PA (min/day)	316.7	(84.2)
Daily LPA (min/day)	292.3	(80.4)
Daily MVPA (min/day)	24.4	(23.2)
Daily long-bout MVPA (min/day)^a^	25.0	(26.9)
Daily step counts (steps/day)	7454.6	(3404.4)
Neighborhood walkability attributes		
Population density (person/km^2^)	30594.3	(14698.4)
Street connectivity (node/m^2^)	211.2	(92.7)
Sidewalk availability (m^2^)	3603.1	(2704.9)
Access to destinations (amount)	14.9	(11.7)
Access to public transportation (amount)	23.0	(18.0)

SD, standard deviation, PA, physical activity; MVPA, moderate-to-vigorous physical activity, LPA, light physical activity.

^a^ Long bouts were defined as bouts lasting ≥10 min.

### Associations between covariates and objectively measured PA

Table 3 shows the associations between covariates and objectively measured PA. Older adults aged under 69 years engaged in significantly more daily PA, MVPA, and long-bout MVPA, and acquired significantly more steps than those aged over 70 years. Older men engaged in significantly more daily MVPA and long bout MVPA than older women, whereas older women engaged in significantly more daily total PA and LPA than older men. In addition, married older adults engaged in significantly more daily MVPA and long-bout MVPA than unmarried older adults. Older adults with no tertiary education spent significantly more time engaged in daily LPA compared with those with tertiary education. Older adults who were employed full time spent significantly more time engaged in daily total PA and LPA than those who were not. No significant differences for all objectively measured PA between the subgroups of living status and BMI were observed.

**Table 3:** Associations between covariates and objectively measured PA.

Variables	Daily Total PA	Daily LPA	Daily MVPA	Daily long-bout MVPA	Daily step counts
	Mean (SD)	Mean (SD)	Mean (SD)	Mean (SD)	Mean (SD)
**Age**					
Under 69 year	332.0 (74.7)	301.2 (73.7)	30.7 (24.1)	30.9 (27.8)	8468.2 (3267.5)
70+ year	294.2 (92.7)	279.1 (88.3)	15.2 (18.5)	17.1 (23.9)	5998.7 (3109.4)
p-value	0.01*	0.13	<0.001**	0.005**	<0.001**
**Gender**					
Men	292.0 (90.3)	255.3 (85.0)	36.7 (27.8)	42.2 (34.6)	8408.9 (4051.7)
Women	326.6 (80.0)	307.1 (74.6)	19.5 (19.3)	18.3 (19.5)	7061.3 (3059.3)
p-value	0.04*	0.001*	<0.001**	<0.001**	0.07
**Marital status**					
Married	312.5 (79.6)	283.6 (776.6)	28.9 (25.1)	29.2 (27.1)	7727.2 (3487.6)
Unmarried	324.9 (92.9)	309.0 (87.4)	15.9 (16.6)	17.6 (25.4)	6916.2 (3245.0)
p-value	0.44	0.09	0.001*	0.02*	0.21
**Living status**					
Living with others	317.0 (84.0)	291.5 (80.4)	25.5 (23.9)	26.3 (27.7)	7569.3 (3446.2)
Living alone	314.5 (88.9)	298.4 (83.1)	16.1 (14.2)	16.0 (18.5)	6455.7 (3088.1)
p-value	0.92	0.73	0.13	0.22	0.27
**Educational level**					
Tertiary education	292.8 (81.2)	260.5 (68.7)	32.3 (24.2)	31.4 (31.3)	8238.7 (4135.9)
No tertiary education	323.2 (84.2)	301.1 (82.2)	22.3 (22.6)	23.5 (25.6)	7233.7 (3177.3)
p-value	0.10	0.01*	0.05	0.18	0.25
**Employment**					
Full-time job	403.2 (92.5)	374.4 (70.5)	28.7 (36.0)	35.8 (50.6)	9472.2 (4655.1)
Non-full time job	313.9 (82.8)	289.6 (80.1)	24.4 (23.0)	24.9 (26.2)	7385.2 (3372.4)
p-value	0.04*	0.04*	0.72	0.70	0.23
**Self-rated health**					
Good	323.8 (70.7)	292.9 (71.0)	31.0 (26.3)	27.3 (25.9)	8221.9 (3397.9)
Poor	314.9 (89.8)	292.1 (84.8)	21.9 (21.5)	24.6 (27.8)	7155.7 (3395.7)
p-value	0.55	0.96	0.04*	0.62	0.12
**BMI**					
Non-overweight	310.6 (87.3)	283.3 (80.0)	27.3 (25.0)	26.0 (27/4)	7824.5 (3709.7)
Overweight	323.2 (81.0)	301.8 (80.3)	21.4 (20.9)	24.5 (26.8)	7068.3 (3066.6)
p-value	0.40	0.20	0.13	0.76	0.22

PA, physical activity; MVPA, moderate-to-vigorous physical activity; BMI, body mass index.

*p < .05

**p < .001

### Neighborhood environmental attributes and objectively measured PA

Table 4 shows the associations between neighborhood environmental attributes and objectively measured PA. After adjusting for potential confounders, we found that sidewalk availability (β = 0.165; 95% confidence interval: 0.006, 0.412; p = 0.043) was positively associated with daily step counts. No significant neighborhood walkability attributes were found to be related to objectively measured PA, including time spent in total PA, LPA, MVPA, long MVPA bout.

**Table 4:** Associations between neighborhood environmental attributes and objectively measured PA in older adults.

Neighborhood environmental attribute	β^a^	95% CI	p
**Total PA**			
Population density	0.012	(-0.001, 0.001)	0.866
Street connectivity	0.036	(-0.096, 0.162)	0.614
Sidewalk availability	0.124	(0.000, 0.008)	0.076
Access to destinations	0.080	(-0.416, 1.559)	0.254
Access to public transportation	0.092	(-0.211, 1.075)	0.186
**MVPA**			
Population density	0.013	(0.000, 0.000)	0.876
Street connectivity	0.015	(-0.038, 0.046)	0.858
Sidewalk availability	0.138	(0.000, 0.003)	0.098
Access to destinations	0.115	(-0.095, 0.548)	0.165
Access to public transportation	0.119	(-0.057, 0.363)	0.151
**LPA**			
Population density	0.009	(-0.001, 0.001)	0.900
Street connectivity	0.034	(-0.091, 0.150)	0.633
Sidewalk availability	0.090	(-0.001, 0.007)	0.190
Access to destinations	0.050	(-0.579, 1.268)	0.461
Access to public transportation	0.063	(-0.323,0.881)	0.360
**Long MVPA bout**			
Population density	0.008	(0.000, 0.000)	0.929
Street connectivity	0.016	(-0.044, 0.054)	0.848
Sidewalk availability	0.074	(-0.001, 0.002)	0.378
Access to destinations	0.079	(-0.193, 0.556)	0.339
Access to public transportation	0.051	(-0.168, 0.322)	0.537
**Steps counts**			
Population density	0.075	(-0.022, 0.056)	0.377
Street connectivity	0.077	(-3.3, 9.0)	0.361
Sidewalk availability	0.165	(0.006, 0.412)	0.043*
Access to destinations	0.115	(-13.3, 80.1)	0.159
Access to public transportation	0.119	(-8.0, 52.9)	0.148

CI, confidence interval; PA, physical activity; MVPA, moderate-to-vigorous physical activity, LPA, light physical activity.

^a^Adjusted for gender, age, marital status, educational level, employment status, living status, self-rated health, BMI, and accelerometer wear time.

*p < .05.

## Discussion

This study fills a research gap by examining the associations between five GIS-derived neighborhood walkability attributes and five metrics of objectively measured PA among community dwelling older adult population. After adjusting for potential confounders, we found that greater sidewalk availability was positively associated with daily step counts in older adults. Nevertheless, because encouraging PA in older adults is a public health priority, this finding has critical implications for urban designers and local policy-makers. Increasing sidewalk availability in the neighborhood would promote PA in older adults.

Among the five neighborhood walkability attributes and five metrics of objectively measured PA, we only observed a positive association between sidewalk availability and daily steps. Although previous studies have found positive associations between availability of sidewalks and walking for exercise [40] or transportation [41], these studies were limited by using self-reported measures of walking. Our finding extends these previous findings and confirms the positive link between sidewalk availability and accelerometer-determined daily steps in the population of older adults. It is possible that roadways with sidewalks in the neighborhood can provide the benefits of safety (i.e., keep people from the traffic and result in less crashes) and mobility (from home to destinations such as retail facilities and open public spaces) for daily PA [42]. In particular, older adults were found to be more sensitive and easily influenced by the neighborhood built environment [8]. Thus, our finding suggest that sidewalks are important urban infrastructure for older adults’ accumulation of daily PA in the neighborhood. Future prospective studies are warranted to deeper understand the long-term impact of sidewalk availability on PA behavior in older adults.

Previous studies have found that objectively measured environmental attributes such as higher residential density and good access to recreational destinations (e.g., access to shops or public transit) are positively associated with objectively measured PA [9,10,12–14]. However, these findings are inconsistent with our own. There are several possible reasons for this inconsistency. First, unlike most previous studies, our study was conducted in the general community and not in a specific setting such as a retirement village [10] or senior living residence [9]. Moreover, the participants in our sample were community-dwelling older adults who were relatively younger (mean age: 69.9 year) and more active (24.4 min MVPA/day) than those of previous studies [9,10,12–14]. The different setting and a sample of younger and more active older adults may explain why our study’s findings were different from those of previous studies.

This study had several strengths. Five metrics of objectively measured PA of older adults, including total PA, LPA, MVPA, long MVPA bouts, and daily step counts in free-living conditions were used, which provides important evidence for modifying PA behaviors in older adults. In addition, neighborhood walkability elements of this study were determined by GIS, which can provide actual attributes of the built environment. Nevertheless, several limitations should be noticed in this study. First, the cross-sectional design of our finding may limit the conclusions that can be drawn because the causality between environmental attributes and PA cannot be assumed. Second, a potential confounder -self-selection, was not considered in the present study. Third, the exposure variables (neighborhood walkability attributes) were calculated by using participants’ self-reported living neighborhood and not exact residential address. This is because reporting personal residential address in detail is a vulnerable matter for the senior of Taiwan [43]. However, residential neighborhood has been used widely as the validated geographic unit for measuring walkability attributes in neighborhoods [44]. In addition, we did not examine the role of different destinations in older adults’ physical activity in this study. Future studies further examining this issue are warranted. Finally, other factors such as weather, terrain, quality of the sidewalks and air pollution in the area, which may affect older adults’ walking behavior were not included in this study.

## Conclusions

Sidewalk availability in the neighborhood may play an important role in the accumulation of daily steps in older adults. Our finding could be provided to aid planners and local policy-makers in the design or re-design of neighborhoods to promote daily steps among older adults. Future studies with larger participants using a prospective design and both subjective and objective measures are warranted to further confirm our results.
